# Exogenous Parathyroid Hormone Alleviates Intervertebral Disc Degeneration through the Sonic Hedgehog Signalling Pathway Mediated by CREB

**DOI:** 10.1155/2022/9955677

**Published:** 2022-02-27

**Authors:** You Li, Yifan Wei, He Li, Hui Che, Dengshun Miao, Cheng Ma, Yongxin Ren

**Affiliations:** ^1^Department of Orthopaedics, The First Affiliated Hospital of Nanjing Medical University, Nanjing, China; ^2^Department of Trauma and Reconstructive Surgery, RWTH Aachen University Hospital, Aachen, Germany; ^3^University Medical Center, Albert-Ludwigs-University, Freiburg, Germany; ^4^The Research Center for Bone and Stem Cells, Department of Anatomy, Histology and Embryology, Nanjing Medical University, Nanjing, China

## Abstract

As an important hormone that regulates the balance of calcium and phosphorus, parathyroid hormone (PTH) has also been found to have an important function in intervertebral disc degeneration (IVDD). Our aim was to investigate the mechanism by which PTH alleviates IVDD. In this study, the PTH 1 receptor was found to be highly expressed in severely degenerated human nucleus pulposus (NP) cells. We found in the mouse model of IVDD that supplementation with exogenous PTH alleviated the narrowing of the intervertebral space and the degradation of the extracellular matrix (ECM) caused by tail suspension (TS). In addition, inflammation, oxidative stress, and apoptosis levels were significantly increased in the intervertebral disc tissues of TS-induced mice, and the activity of NP cells was decreased. TS also led to the downregulation of Sonic hedgehog (SHH) signalling pathway-related signal molecules in NP cells such as SHH, Smoothened, and GLI1. However, supplementation with exogenous PTH can reverse these changes. *In vitro*, PTH also promotes the activity of NP cells and the secretion of ECM. However, the antagonist of the SHH signalling pathway can inhibit the therapeutic effect of PTH on NP cells. In addition, a cAMP-response element-binding protein, as an important transcription factor, was found to mediate the promotion of PTH on the SHH signalling pathway. Our results revealed that PTH can alleviate IVDD by inhibiting inflammation, oxidative stress, and apoptosis and improving the activity of NP cells via activating the SHH signalling pathway.

## 1. Introduction

Degenerative disc disease is one of the most common musculoskeletal disorders and a leading cause of disability [1, 2]. The intervertebral disc consists of the annulus fibrosus (AF) on the exterior border of the disc, the nucleus pulposus (NP) in the interior, and endplates [3]. The NP contains the extracellular matrix (ECM), including collagen II, proteoglycan, and NP cells [3]. The biological mechanism of intervertebral disc degeneration (IVDD) is unclear. Current studies have shown that these factors including ECM degradation, inflammation, oxidative stress, and decreased activity of NP cells are important in inducing IVDD [4–6].

The hedgehog signalling pathway is composed of receptors and ligands named hedgehog proteins, which are secreted proteins, including Sonic hedgehog (SHH), Indian hedgehog (IHH), and Desert hedgehog (DHH) [7, 8]. Both SHH and IHH produce secreted proteins that bind to the transmembrane protein PATCH&ED (PTCH), thereby activating the hedgehog signalling pathway [7]. The activated hedgehog signalling pathway binds to PTCH and leads to the disinhibitory effect of PTCH on Smoothened (SMO) [7]. Downstream signals cause hyperphosphorylation of costal-2 and Fu and activate the GLI family (GLI1, GLI2, and GLI3) of transcription factors [7]. GLI then enters the nucleus in a full-length form to activate the transcription of downstream target genes, which changes the biological characteristics of the cells [7]. NP cells originate from the embryo's notochord; SHH is essential for the formation of the notochord sheath and embryonic NP cells [9]. Previous studies have found that SHH is abundantly expressed in the intervertebral discs of neonatal mice, especially in NP cells [10, 11]. The SHH signalling pathway has been identified as playing an important role in regulating the proliferation and differentiation of NP cells during the development of intervertebral discs in mice [11].

Parathyroid hormone (PTH) is an important hormone secreted by the parathyroid glands to regulate bone remodelling and calcium homeostasis [12]. Normally, parathyroid hormone binds to the G protein-coupled receptor—parathyroid hormone 1 receptor (PTH1R)—to activate adenylate cyclase, which then activates the second messenger, cyclic adenosine-3′,5′-monophosphate (cAMP) and then activates protein kinase A (PKA) [13]. PKA can phosphorylate the transcription factor named cAMP-response element-binding protein (CREB). Phosphorylated CREB regulates downstream target genes to play a corresponding role [13]. Previous studies have shown that PTH can attenuate articular cartilage degeneration and promote cartilage regeneration [14, 15]. Recent studies have shown that, in addition to promoting bone remodelling, PTH also has a protective effect on intervertebral discs [16, 17]. Jia et al. found that PTH (1–34) can increase the levels of proteoglycan and collagen II in the intervertebral discs of ovariectomized rats and alleviate IVDD [16]. Madiraju et al. revealed that PTH can promote the release of high concentrations of Ca^2+^ from NP cells to the outside of the cells to reduce the intracellular Ca^2+^ concentration and prevent NP cells from calcification. As a result, they found that PTH could reduce damage in NP cells by promoting the expression of collagen II and reduce the expression of collagens I and X [17]. Therefore, we speculated that PTH could maintain the homeostasis of intervertebral discs and could have great potential for the treatment of degenerative disc diseases. However, the cellular and molecular mechanisms remain unclear.

Our previous research found that PTH can activate the related molecules of the hedgehog signalling pathway, thus promoting chondrocyte proliferation. For instance, IHH could promote the proliferation and indirectly inhibit chondrocyte differentiation through the parathyroid hormone-related protein- (PTHrP-) dependent pathway or the PTHrP-independent pathway. Given that studies have shown that NP cells express PTH1R rather than PTHrP [18], we suspected that PTH may alleviate the degeneration of NP cells through a PTHrP-independent pathway by activating the SHH signalling pathway. We used C57BL/6 mice to make a model of IVDD and recombinant human PTH (1–34) was used to treat the mice. The effect of PTH on the NP tissues of mice was clarified by detecting the ECM expression, the level of inflammation and oxidative stress, proliferation ability, and the SHH signalling pathway. In addition, we verified the effect of PTH *in vitro*.

## 2. Materials and Methods

### 2.1. Human NP Collection

Thirty-two human NP tissues were collected from patients who had undergone discectomy at the First Affiliated Hospital of Nanjing Medical University. Patients with IVDD were graded according to the Pfirrmann grade (based on Magnetic Resonance Imaging), including 6 patients with Grade 2 (G2), 11 patients with G3, 8 patients with G4, and 7 patients with G5. A higher score indicates more serious degeneration. The informed consent of the patients was obtained before the operation. This project was implemented with the approval of the Ethics Committee of the First Affiliated Hospital of Nanjing Medical University (registered number: 2018 SR-233).

### 2.2. Human NP Cell Isolation and Culture

The procedure for isolating NP cells from human intervertebral discs was the same as our previous study [19]. Dulbecco's modified Eagle media are as follows: Nutrient Mixture F-12 (Gibco, USA) containing 10% foetal bovine serum (Gibco, USA) and 1% penicillin plus streptomycin (Invitrogen, USA) was used to culture human NP cells. Recombinant human IL-1*β* (Liankebio, China) was used to induce the degeneration of the NP cells. Purmorphamine (PUA, Selleck Chemicals, USA) and cyclopamine (CYA, Selleck Chemicals, USA) acting on SMO were used to activate or inhibit the SHH signalling pathway.

### 2.3. Animals and IVDD Model

Sixty 3-month-old male C57BL/6 mice were used in this study. Animal use was approved by the Institutional Animal Care and Use Committee of Nanjing Medical University (approval number: IACUC-1709021). After the mice grew to 3 months old, we anesthetized mice using 0.3% sodium pentobarbital (0.2 mL/10 g) and suspended the tails of the mice for 4 weeks in special cages where the mice were free to eat to make the IVDD model. The height of the suspension was controlled so that the mice's hind legs were just off the ground [19]. Four weeks later, we detected the mice's spines via X-rays and collected mouse spines for subsequent experiments. Mice were given low- (20 *μ*g/kg) or high- (40 *μ*g/kg) concentration recombinant human PTH; the mice were categorized into the low-concentration PTH (LPTH) group and the high-concentration PTH (HPTH) group, respectively. During the modelling, PTH was injected into the mice by subcutaneous injection at 10 : 00 daily [20].

### 2.4. Radiological Study

The mice were placed in the right decubitus position in a small animal X-ray digital imager (RS 2000 Pro, RADSOURCE, USA) for X-ray imaging (T13–L6). The disc height index (DHI) was used to analyse the X-ray results [19]. DHI is the ratio of the height of the intervertebral space to the height of the two adjacent vertebral bodies.

### 2.5. Histological Staining

The human NP tissues and mouse lumbar spines (L3–L6) were placed in 4% paraformaldehyde fixative (NCM Biotech, China) for 24 h and placed in the decalcification solution for 1 week. The lumbar spines of the mice were then paraffin-encased and sliced into 5 *μ*m sections. The slices were baked at 37°C for 3 days. HE staining was used to observe the morphology of the intervertebral discs. We also detected the expression of ECM through Safranin O staining and Masson staining in the human intervertebral discs [19]. The proteoglycan content ratio of the human NP tissues was approximated by the quantification of the Safranin O staining intensity (SOI) [21, 22]. Collagen volume fraction (COV) was used to quantify the Masson staining [23]. COV refers to the percentage of collagen area (blue area) to total tissue area.

### 2.6. Immunohistochemical (IHC) Staining

The mouse lumbar spines (L3–L6) were used for IHC staining. After deparaffinization and hydration, the slices were placed in the citrate buffer and heated to 95°C for 10 min; 3% H_2_O_2_ was then used to inactivate peroxidases. After blocking nonspecific antigens with 10% goat serum, we incubated the slices with primary antibody dilution solution at 4°C overnight. The primary antibodies used were as follows: PTH1R (1 : 100, ab75150, Abcam, USA), collagen II (1 : 300, ab34712, Abcam, USA), collagen X (1 : 100, ab58632, Abcam, USA), superoxide dismutase 1 (SOD1, 1 : 1000, ab51254, Abcam, USA), SOD2 (1 : 200, ab68155, Abcam, USA), proliferating cell nuclear antigen (PCNA, 1 : 300, ab92552, Abcam, USA), Ki67 (1 : 500, ab15580, Abcam, USA), caspase3 (1 : 1000, ab184787, Abcam, USA), caspase9 (1 : 300, ab202068, Abcam, USA), SHH (1 : 500, ab135240, Abcam, USA), and GLI1 (1 : 200, ab217326, Abcam, USA). The slices were then washed 3 times with phosphate buffer solution (PBS) and incubated with secondary antibody dilution (Goat Anti-Rabbit IgG, 1 : 500, 12-348, Sigma Aldrich, USA; Goat Anti-Mouse IgG, 1 : 500, 12-349, Sigma Aldrich, USA) at room temperature for 1 h. Diaminobenzidine was used to develop the colour. Finally, the cell nuclei were stained with haematoxylin (Beyotime, China), and the slices were mounted with neutral gum (Beyotime, China) after dehydration.

### 2.7. Protein Extraction and Western Blot

The RIPA lysis buffer (Beyotime, China) was used to extract total protein from the mouse intervertebral discs (L3–L6) and human NP cells. Western blot was implemented to detect the expression of certain proteins in the previously mentioned tissues and cells. Equal amounts of proteins were separated by 10% sodium dodecyl sulfate polyacrylamide gel electrophoresis and transferred onto a polyvinylidene fluoride (PVDF) membrane (Millipore, USA). After blocking with 5% bovine serum albumin (Sigma Aldrich, USA), the PVDF membrane was incubated with the following primary antibodies: *β*-actin (1 : 3000, ab8226, Abcam, USA), collagen II (1 : 300, ab58632, Abcam, USA), aggrecan (1 : 1000, ab3778, Abcam, USA), collagen X (1 : 300, ab58632, Abcam, USA), interleukin 1 beta (IL-1*β*, 1 : 3000, ab254360, Abcam, USA), SOD1 (1 : 50000, ab51254, Abcam, USA), SOD2 (1 : 1000, ab68155, Abcam, USA), PCNA (1 : 1000, ab92552, Abcam, USA), caspase3 (1 : 2000, ab184787, Abcam, USA), caspase8 (1 : 2000, ab108333, Abcam, USA), caspase9 (1 : 2000, ab202068, Abcam, USA), Bcl2 (1 : 2000, ab182858, Abcam, USA), SHH (1 : 1000, ab135240, Abcam, USA), SMO (1 : 1000, ab236465, Abcam, USA), and GLI1 (1 : 1000, ab217326, Abcam, USA). The secondary antibody (Goat Anti-Rabbit IgG, 1 : 5000, ab6721, Abcam, USA; Goat Anti-Mouse IgG, 1 : 5000, ab6789, Abcam, USA) was used to probe with the above-mentioned primary antibody. Immunoreactive bands were visualized with electrochemiluminescence (Millipore, USA) and analysed using ImageJ 2.1.0 (National Institutes of Health, USA).

### 2.8. RNA Extraction and Quantitative Real-Time PCR (RT-PCR)

The TRIzol reagent (Invitrogen, USA) was used to extract total RNA from mouse intervertebral discs (L3–L6) and human NP cells. cDNAs were synthesized using the SuperScript IV reverse transcriptase (Thermo Fisher, USA). The SYBR Green Master Mix (Vazyme, China) was used to amplify specific sequences of cDNAs from specific sequences. GAPDH was used for normalisation; 2^-*ΔΔ*CT^ was used to represent the relative expression of mRNA. The RT-PCR primers are shown in Table 1.

### 2.9. Enzyme-Linked Immunosorbent Assay (ELISA)

Mouse intervertebral disc tissues (L3–L6) and NP cells supernatant were collected for ELISA testing. Mouse intervertebral disc tissues were scraped with a sterile blade and lysed with RIPA lysis (Beyotime, China). Concentrations of IL-1*β* and tumour necrosis factor- (TNF-) *α* in the intervertebral disc tissues and NP cells supernatant were detected with ELISA kits (Liankebio, China) used in accordance with the manufacturer's instructions.

### 2.10. Flow Cytometry Analyses

Apoptosis levels of NP cells were detected by flow cytometry. An Annexin V/PI kit (KeyGen, China) was used to detect the apoptosis levels of NP cells in accordance with the manufacturer's instructions. Annexin V was used to bind phosphatidylserine to apoptotic cells in order to distinguish apoptotic cells from normal cells. Propidium iodide can penetrate the membranes of late apoptotic and dead cells and was therefore used to differentiate between early and late apoptotic cells. Flow cytometry (A28997; Thermo Fisher Scientific) was used to detect apoptosis with the Attune NxT Software.

### 2.11. Immunofluorescence (IF) Staining

The NP cells were immobilized with 4% paraformaldehyde fixative for 15 min and immersed in 0.1%Triton X-100 for 20 min. After blocking with 10% goat serum for 1 h, NP cells were incubated with primary antibody dilution (collagen II, 1 : 300, ab34712, Abcam, USA; collagen X, 1 : 100, ab58632, Abcam, USA) at 4°C overnight. The next day, fluorescent secondary antibody dilution (Goat Anti-Rabbit IgG Alexa Fluor® 488, 1 : 500, ab150077, Abcam, USA) was used in the dark for 1 h, and the cell nuclei were stained using DAPI (Sigma Aldrich, USA). Finally, we used a fluorescence microscope (Leica, Germany) to observe the staining results.

### 2.12. Cell Counting Kit-8 (CCK8) Cell Viability Assay

CCK8 was used to determine the effects of PTH, CYA, and PUA on the viability and proliferation of NP cells. After adding 10 *μ*L CCK8 reagent (Dojindo, Japan) to each well of a 96-well plate after 6h, 12 h, 24 h, and 48 h of stimulation, the NP cells were incubated at 37°C for 2 h. A microplate reader measured the absorbance at 450 nm in the dark. NP cells were divided into blank, control, and experimental groups. The blank group had only medium and no cells, while the control group had cells without drug stimulation. Cell viability = (OD experimental–OD blank)/(OD control–OD blank).

### 2.13. 5-Ethynyl-2'-deoxyuridine (EdU) Cell Proliferation Assay

The EdU cell proliferation assay determined the effect of PTH, CYA, and PUA on the proliferation of human NP cells. EdU is a thymine nucleoside analogue that can replace thymine during cell proliferation into replicating DNA. The proliferation of human NP cells was measured with an EdU cell proliferation kit (Solarbio, China) in accordance with the manufacturer's instructions.

### 2.14. Chromatin Immunoprecipitation (ChIP) Assay

The binding region of the transcription factor CREB to the SHH promoter was certified with the ChIP assay. Using the National Center for Biotechnology Information database (http://www.ncbi.nlm.nih.gov/), we searched for SHH promoter regions (2000-bp area). The JASPAR Core Database (http://jaspar.genereg.net/) was used to predict possible binding sites. The primers were constructed by Shanghai Generay Biotech. ChIP assays were performed using a ChIP kit (MilliporeSigma, USA) according to the manufacturer's instructions. The relative binding of SHH to CREB was assessed with PCR, followed by digital imaging of agarose gels. Lipofectamine 3000 (Thermo Fisher, USA) was used for cell transfection.

### 2.15. Plasmid Constructs and Dual Luciferase (Luc) Reporter Gene Assay

Negative control (NC), wild type (WT), and mutant (MUT) SHH gene promoter segments were synthesized by GeneChem (China) and cloned into the PGL4.23-basic Luc vector. Human NP cells were divided into 6 groups: NC+Promoter-NC-Luc, NC+Promoter-WT-Luc, NC+Promoter-MUT-Luc, CREB+Promoter-NC-Luc, CREB+Promoter-WT-Luc, and CREB+Promoter-MUT-Luc. A dual Luc reporter gene assay was implemented after 48 h of transfection. The ratio of the Firefly Luc value to the Renilla Luc value represented the relative expression level of Luc.

### 2.16. Statistical Analyses

The SPSS software (version 21.0, USA) was used to analyse all the results in this study. All data are expressed as mean ± standard deviation. The Shapiro-Wilk normality test was used to assess data distribution. One-way ANOVA was used to compare differences between groups followed by post hoc testing (Tukey) when data are normally distributed. If not, the Mann–Whitney test was used. *p* < 0.05 was considered statistically significant. All charts were generated using GraphPad Prism 7.0 (GraphPad Software, USA).

## 3. Results

### 3.1. PTH1R Was Upregulated in the NP Tissues of Degenerated Human Intervertebral Discs

**Figure 1:**
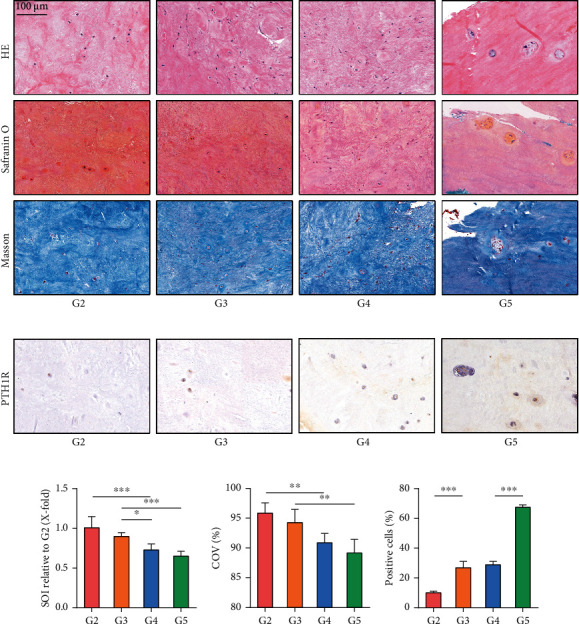
The correlation between PTH1R expression and the degree of intervertebral disc degeneration. Human intervertebral disc tissues were collected from the Pfirrmann grade G2–G5 groups. (a) HE staining showed the morphology of all NP tissues. Safranin O-stained NP cells orange and fibres blue. Masson-stained NP cells blue and fibres red. Magnification: ×200. (b) IHC staining of PTH1R in human NP tissues. Magnification: ×200. (c) Quantitative analysis of Safranin O staining. (d) Quantitative analysis of Masson staining. (e) Quantitative analysis of IHC staining. Data are presented as mean ± SD. ^∗^*p* < 0.05; ^∗∗^*p* < 0.01; ^∗∗∗^*p* < 0.001.

To clarify the correlation between PTH/PTH1R and IVDD, the expression of PTH1R was detected in human NP tissues with different degrees of degeneration. Human NP tissues were classified into G2, G3, G4, and G5 groups according to the Pfirrmann grade (G1 was not included due to only mild degeneration). HE staining showed that the structure of NP tissues loosened and the number of NP cells decreased while multinucleated giant cells appeared with higher Pfirrmann grades. Safranin O staining and Masson staining showed that there were fewer proteoglyphages and collagen fibres in severely degenerated discs (Figures 1(a), 1(c), and 1(d)). IHC staining showed that PTH1R was expressed more in the NP tissues with higher Pfirrmann grades (Figures 1(b) and 1(e)). These results indicate that the expression of PTH1R was positively correlated with the degree of IVDD.

### 3.2. PTH Partially Alleviated IVDD in Mice

**Figure 2:**
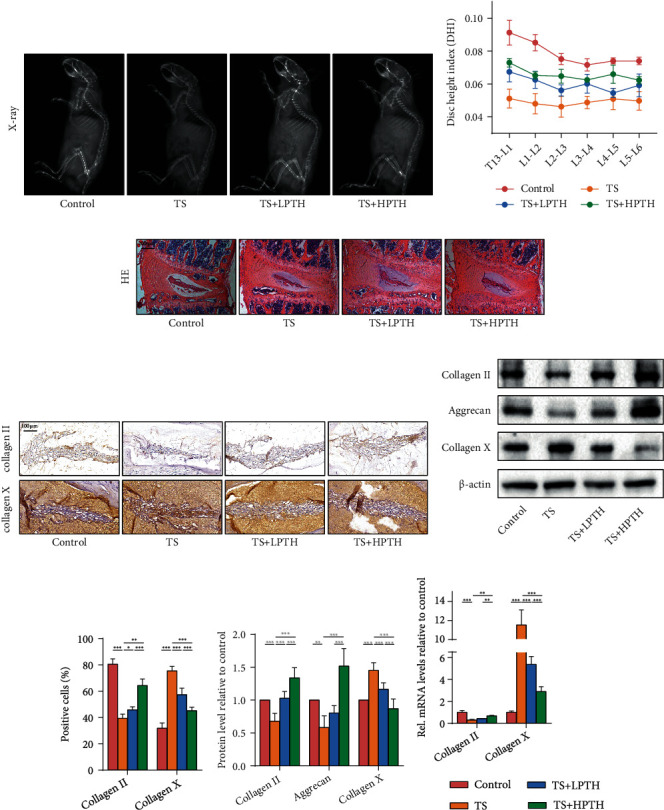
The effect of exogenous PTH on the structure and ECM of mouse intervertebral discs. (a) X-ray images of mice in the right decubitus position. (b) DHI according to X-ray images. (c) HE staining of mouse intervertebral discs. Magnification: ×100. (d) IHC staining of collagen II and collagen X of mouse intervertebral discs. Magnification: ×200. (e) The protein levels of collagen II, aggrecan, and collagen X of mouse intervertebral discs. (f) Quantitative analysis of IHC staining. (g) Quantitative analysis of western blot. (h) The mRNA levels of collagen II and collagen X of mouse intervertebral discs. Data are presented as mean ± SD (*n* = 6). ^∗^*p* < 0.05; ^∗∗^*p* < 0.01; ^∗∗∗^*p* < 0.001.

To further investigate the effect of PTH/PTH1R on IVDD, we used C57/BL6 mice to establish IVDD models by the tail suspension (TS) method and treated mice with two different concentrations of PTH solution. Compared with the control group, X-rays showed that the DHI of the TS group was lower, while PTH improved the structure of the intervertebral discs; a high concentration of PTH had better efficacy (Figures 2(a) and 2(b)). HE staining showed that the intervertebral space height of the TS group was lower than that of the normal group and the morphology of the NP tissues was compressed, whereas the PTH group improved (Figure 2(c)). IHC staining (Figures 2(d) and 2(f)) and western blot (Figures 2(e) and 2(g)) showed that the collagen II and aggrecan of intervertebral discs in the TS group significantly decreased and collagen X showed a compensatory increase while PTH reversed it. PTH can also promote collagen II mRNA expression and reduce collagen X mRNA expression (Figure 2(h)). These results demonstrated that supplementation with exogenous PTH suppressed the progression of IVDD in mice.

### 3.3. PTH Reduced the Levels of Inflammation, Oxidative Stress, and Apoptosis in Mouse Intervertebral Discs and Promoted NP Cell Proliferation

**Figure 3:**
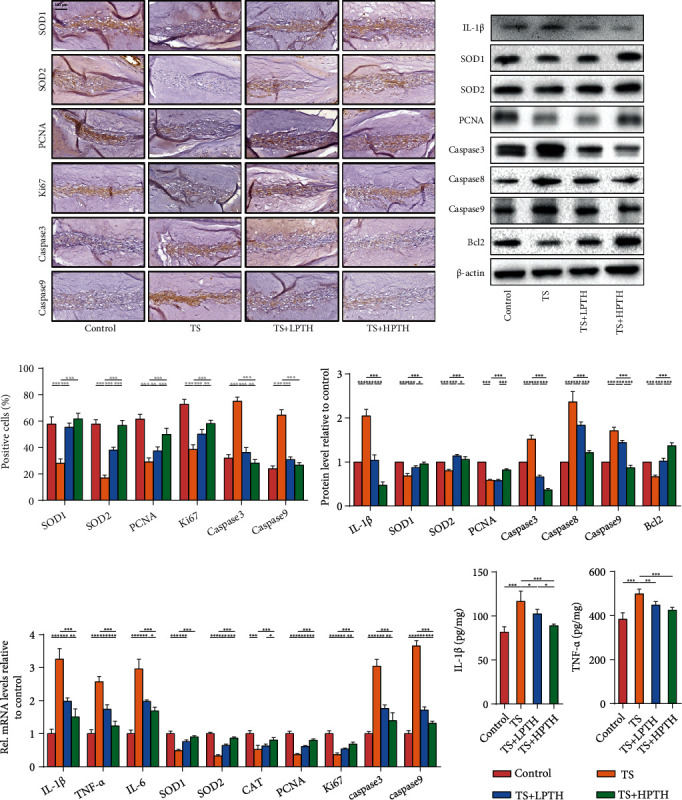
The effect of exogenous PTH on inflammation, oxidative stress, apoptosis, and proliferation of the mouse intervertebral discs. (a) IHC staining of SOD1, SOD2, PCNA, Ki67, caspase3, and caspase9 of mouse intervertebral discs. Magnification: ×200. (b) The protein levels of IL-1*β*, SOD1, SOD2, PCNA, caspase3, caspase8, caspase9, and Bcl2 of mouse intervertebral discs. (c) Quantitative analysis of IHC staining. (d) Quantitative analysis of western blot. (e) The mRNA levels of IL-1*β*, TNF-*α*, IL-6, SOD1, SOD2, catalase (CAT), PCNA, Ki67, caspase3, and caspase9 of mouse intervertebral discs. (f) ELISA of IL-1*β* and TNF-*α* of mouse intervertebral discs. Data are presented as mean ± SD (*n* = 6). ^∗^*p* < 0.05; ^∗∗^*p* < 0.01; ^∗∗∗^*p* < 0.001.

The above results suggested that exogenous PTH improves the stability of the ECM of the intervertebral discs, so we examined the effect of PTH on the physiological and pathological status of NP cells. IHC staining (Figures 3(a) and 3(c)) and western blot (Figures 3(b) and 3(d)) showed that SOD1, SOD2, PCNA, and Ki67 in the TS group of the mouse intervertebral discs decreased, indicating lessened antioxidant and proliferation ability. In addition, the expression of caspase3, caspase8, and caspase9 in the TS group increased, while that of Bcl2 decreased. When supplemented with exogenous PTH, the corresponding indicators of the intervertebral discs were significantly changed, indicating that PTH ameliorated the levels of oxidative stress and apoptosis and increased the proliferation of cells. The same conclusions were found at the mRNA level (Figure 3(e)). In addition, we measured the changes in inflammatory factors in mouse intervertebral discs by western blot (Figures 3(b) and 3(d)), RT-PCR (Figure 3(e)), and ELISA (Figure 3(f)). PTH effectively reduced the expression of inflammatory factors (IL-1*β*, IL-6, and TNF-*α*) in the mouse intervertebral disc.

### 3.4. PTH Promotes SHH Signalling Pathway Activity in Mouse Intervertebral Discs

**Figure 4:**
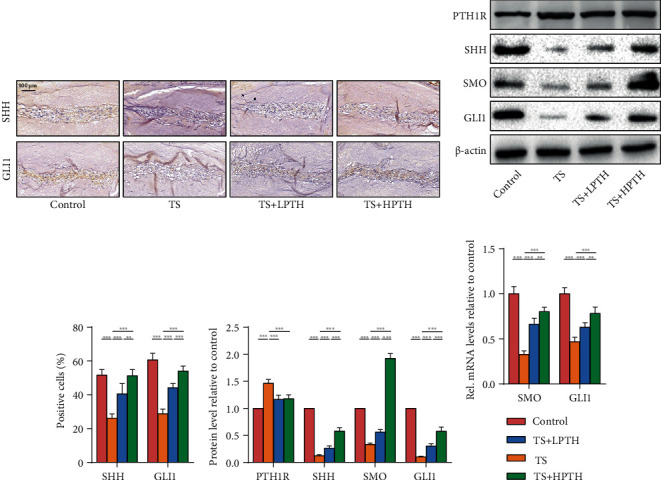
The effect of exogenous PTH on the SHH signalling pathway of the mouse intervertebral discs. (a) IHC staining of SHH and GLI1 of mouse intervertebral discs. Magnification: ×200. (b) The protein levels of PTH1R, SHH, SMO, and GLI1 of mouse intervertebral discs. (c) Quantitative analysis of IHC staining. (d) Quantitative analysis of western blot. (e) The mRNA levels of SMO and GLI1 of mouse intervertebral discs. Data are presented as mean ± SD (*n* = 6). ^∗^*p* < 0.05; ^∗∗^*p* < 0.01; ^∗∗∗^*p* < 0.001.

The SHH signalling pathway is an important pathway in the development of intervertebral discs. In order to determine whether PTH influences the SHH signalling pathway, we detected changes in the expression of key signalling molecules of the SHH signalling pathway in mouse intervertebral discs. The expression levels of SHH, SMO, and GLI1 in TS mice were decreased when measured by IHC staining (Figures 4(a) and 4(c)), western blot (Figures 4(b) and 4(d)), and RT-PCR (Figure 4(e)), which indicated that IVDD was accompanied by decreased activity of the SHH signalling pathway in NP, while the protein and mRNA expression of signal molecules related to the SHH signalling pathway increased after PTH treatment. In addition, we also examined the changes in PTH1R in mice intervertebral discs (Figures 4(b) and 4(d)). Intervertebral discs in mice with IVDD expressed more PTH1R, and the expression of PTH1R decreased after PTH treatment. The results were consistent with human intervertebral disc samples.

### 3.5. PTH Alleviates IL-1*β*-Induced Damage to NP Cells *In Vitro*

**Figure 5:**
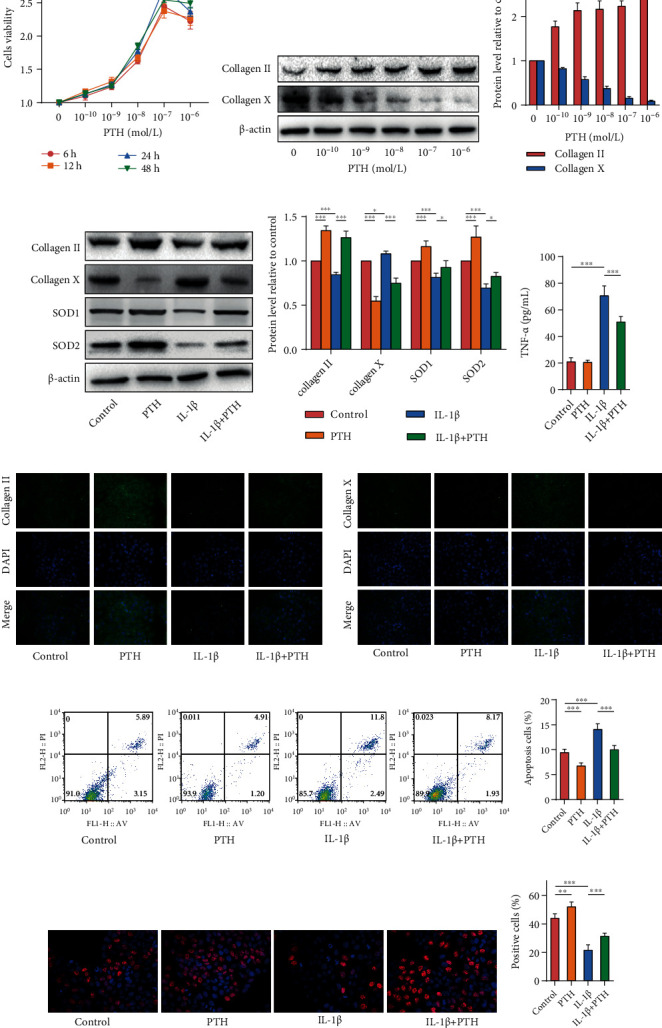
The *in vitro* effect of exogenous PTH on human NP cells. (a) The effect of different concentrations of PTH on NP cell viability. (b) The protein levels of collagen II and collagen X in human NP cells stimulated with different concentrations of PTH. (c) Quantitative analysis of western blot. (d) The protein levels of collagen II, collagen X, SOD1, and SOD2 in human NP cells stimulated with PTH (10^−7^ mol/L) and IL-1*β* (10 ng/mL). (e) Quantitative analysis of western blot. (f) ELISA of TNF-*α* in NP cells supernatant. (g) IF staining of collagen II and collagen X in human NP cells. Magnification: ×200. (h, i) Cell apoptosis levels of human NP cells measured by flow cytometry. (j, k) Cell proliferation levels of human NP cells measured by the EdU cell proliferation assay. Data are presented as mean ± SD (*n* = 6). ^∗^*p* < 0.05; ^∗∗^*p* < 0.01; ^∗∗∗^*p* < 0.001.

We isolated and cultured human primary NP cells for further research on the impact of exogenous PTH on NP cells. The CCK8 assay was conducted to determine the optimal concentration of PTH, which showed that 10^–7^ mol/L PTH for 24 h stimulated NP cells and achieved optimal activity; we thus used 10^–7^ mol/L PTH for 24 h in the subsequent cell experiments (Figure 5(a)). The effects of different concentrations of PTH on the secretion of collagen in NP cells detected by western blot showed that PTH promoted collagen II and inhibited collagen X in a concentration-dependent manner (Figures 5(b) and 5(c)). IL-1*β* (10 ng/mL) was used to induce damage and degeneration of NP cells [19]. Western blot (Figures 5(d) and 5(e)) and IF staining (Figure 5(g)) clearly illustrated that IL-1*β* could induce the degradation of collagen II and increase collagen X in NP cells when compared with control levels, while the intervention of PTH could reverse this process. IL-1*β* also reduced the expression of SOD1 and SOD2 *in vitro*, while PTH could increase their expression. ELISA revealed that PTH could reduce IL-1*β*-induced inflammation *in vitro* (Figure 5(f)). Additionally, PTH could inhibit IL-1*β*-induced apoptosis of NP cells measured by flow cytometry (Figures 5(h) and 5(i)). Similarly, EdU cell proliferation assay showed that PTH could increase the proliferation ability of NP cells (Figures 5(j) and 5(k)).

### 3.6. PTH Regulated NP Cells through the SHH Signalling Pathway

**Figure 6:**
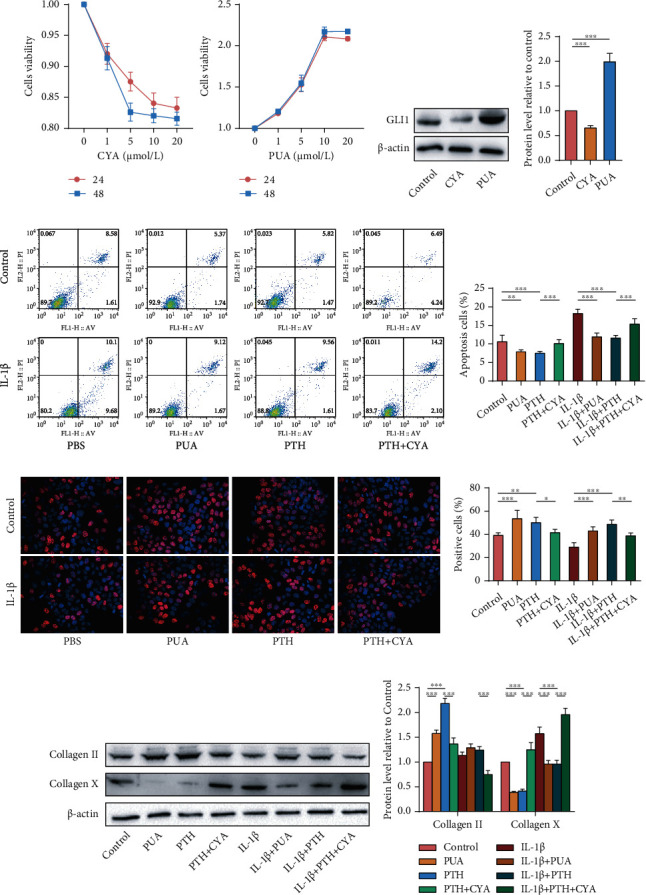
The effect of SHH signalling pathway antagonist (CYA) and agonist (PUA) on human NP cells. (a) The effect of different concentrations of CYA and PUA on the NP cells viability. (b, c) The protein levels and relative quantitative analysis of GLI1 in human NP cells stimulated with CYA and PUA. (d, e) Cell apoptosis levels of human NP cells measured by flow cytometry. (f, g) Cell proliferation levels of human NP cells measured by EdU cell proliferation assay. (h, i) The protein levels and relative quantitative analysis of collagen II and collagen X in human NP cells. Data are presented as mean ± SD (*n* = 6). ^∗^*p* < 0.05; ^∗∗^*p* < 0.01; ^∗∗∗^*p* < 0.001.

To uncover whether PTH retards IVDD by activating the SHH signalling pathway, we used the SHH signalling pathway antagonist CYA and agonist PUA to stimulate NP cells. The optimal concentrations of CYA and PUA were determined by CCK8 assay, and 10 *μ*mol/L for 24 h was chosen (Figure 6(a)). Western blot proved the impact of CYA and PUA on the SHH signalling pathway, showing that CYA could inhibit and PUA could promote the protein expression of GLI1 in the SHH signalling pathway (Figures 6(b) and 6(c)). Flow cytometry (Figure 6(d) and 6(e)) and EdU cell proliferation assay (Figures 6(f) and 6(g)) were performed as mentioned above, and the results revealed that PUA and PTH could inhibit the apoptosis of NP cells and promote cell proliferation, while CYA could do the opposite. As shown by western blot, the collagen II expression after the treatment of PUA and PTH was higher than that in the control group, and the expression of collagen II in the PTH+CYA group was lower than that in the PTH group, whether at the basic level or under IL-1*β* stimulation. Collagen X went in the opposite direction (Figures 6(h) and 6(i)). These results indicate that the SHH signalling pathway played a protective role and that inhibition of the SHH signalling pathway suppressed the protective effect of PTH on NP cells.

### 3.7. CREB Promotes SHH Expression in NP Cells by Activating the SHH Promoter

**Figure 7:**
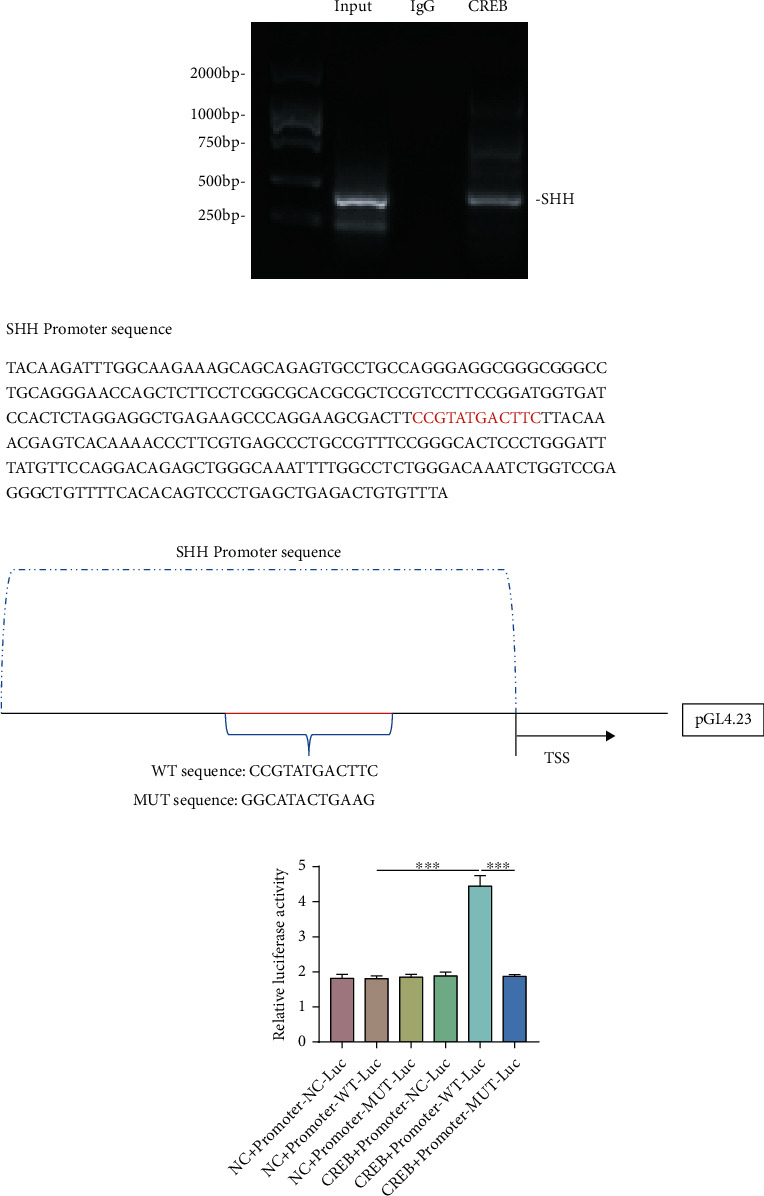
CREB in the PTH/PTH1R signalling pathway bound the promoter sequence of SHH and promoted SHH expression. (a) The immunoprecipitates bound by the CREB antibody were used for PCR. (b) The SHH promoter sequence was as shown. The part marked in red is the predicted binding site. WT and relative mutated sequences were constructed with pGL4.23. (c) The relative Luc activity derived by NC, WT, and MUT-SHH promoter sequences in human NP cells. Data are presented as mean ± SD (*n* = 6). ^∗∗∗^*p* < 0.001.

The above results indicate that the PTH signalling pathway can alleviate NP cell degeneration through the SHH signalling pathway. In order to clarify the mechanism between PTH signalling and the SHH signalling pathway, we found a putative binding site between the transcription factor CREB in the PTH signalling pathway and the SHH promoter region through the JASPAR database. We then verified this possible site through ChIP assay. The product of immunoprecipitation of anti-CREB and irrelevant negative control IgG was used to amplify the SHH promoter fragments. The results of agarose gel electrophoresis showed that the fluorescence intensity of the PCR band of the immunoprecipitation products by anti-CREB antibody was between the Input group and the IgG group, indicating that the anti-CREB antibody binds the upstream promoter of SHH (Figure 7(a)). The SHH promoter sequence is shown in Figure 7(b). We then determined the regulation of CREB on SHH expression through dual Luc reporter assay. WT and the corresponding mutant SHH promoter sequence were inserted into the PGL4.23-basic Luc vectors. Different plasmids were then transfected into human NP cells. There was no significant difference in Luc activity among the three groups without transfection of CREB-overexpressed plasmid. The fluorescence intensity of the CREB+Promoter-WT-Luc group was significantly higher than that of the CREB+Promoter-NC-Luc group, suggesting that CREB successfully activated the SHH promoter. In addition, the cloning of the inactive MUT-SHH sequence did not cause an increase in fluorescence intensity, which confirmed that WT-SHH was the key to promoting the activation of the SHH promoter (Figure 7(c)). These results suggest that CREB may be a crucial mechanism for the PTH/PTH1R signalling pathway to promote SHH transcription in human NP cells.

## 4. Discussion

IVDD is the consequence of multiple factors, including biomechanics, oxidative stress, apoptosis, inflammatory stress, and cell senescence [5]. PTH, as an essential hormone that regulates the balance of calcium and phosphorus, has been found to have other physiological functions in recent years [24, 25]. Caruso et al. [26] used PTH (1–34) in various acute and chronic inflammatory models and found that PTH (1–34) had an inhibitory effect in almost all inflammatory models. In addition, Cheng et al. [27] discovered that PTH1R activation can reduce vascular oxidative stress in diabetic mouse models. Our study also revealed that PTH played an important role in an anti-inflammatory, antioxidant, and antiapoptotic manner in an IVDD mouse model, which may be related to the interaction between the PTH signalling pathway and SHH signalling pathways.

PTH may work by binding to receptors; there are two types of PTH receptors at present, namely PTH1R and PTH2R, both of which are G protein-coupled receptors [28]. PTH2R is mainly distributed in the brain, and its mechanism is unclear [29]. PTH1R is found mainly in bone, the kidneys, and the small intestine and regulates calcium and phosphorus balance [30]. At the cellular level, PTH1R promotes the proliferation, survival, and differentiation of a variety of cells, including osteoblasts, chondrocytes, osteocytes, and some cancer cells [13, 18, 31]. In addition, recent studies have found the expression of PTH1R in intervertebral disc NP cells [32]. Zheng et al. [32] proved that PTH1R was expressed in both NP and AF and confirmed that PTH1R was expressed in notochord-derived NP cells. The high expression of PTH1R in NP cells of aged rat and human intervertebral discs indicated that the PTH pathway may play an important role in maintaining disc homeostasis in response to mechanical load, especially during the aging process. Differing from the focus of previous research, we collected human intervertebral disc NP tissues with various Pfirrmann grades and further demonstrated the degree of NP degeneration by HE staining, Safranin O staining, and Masson staining. Finally, we found that the expression of PTH1R was positively correlated with the level of IVDD.

In order to determine whether PTH is involved in the regulation of IVDD, we established an IVDD model using the TS method in male C57/BL6 mice. As shock absorbers for the spine, the discs' basic role is to distribute load and dissipate energy. Mechanical stress is one of the mechanisms that accelerate the aging process of the discs. Therefore, we chose the TS method to simulate the mechanical change process of aging disc degeneration [19]. Simulating weightlessness with TS changes disc flexion and extension, axial rotation, lateral bending, and hydrostatic pressure, leading to ECM destruction, inflammatory response, and catabolic processes. This process represents the progression of IVDD associated with premature aging [33, 34].

The integrity of the ECM plays an important part in maintaining the normal condition of intervertebral discs and the survival of intervertebral disc cells such as AF cells, NP cells, and notochord cells. Homeostasis in intervertebral discs requires a dynamic balance between the synthesis and degradation of ECM macromolecules [35]. Thus, an imbalance in catabolism and anabolism of ECM may lead to IVDD, where intervertebral disc cells usually undergo a variety of biological changes, including changes in NP cell morphology and phenotype, increases in cell density and apoptosis, which is characterized by the decrease of ECM synthesis ability, and the enhancement of catabolism. In addition, ECM degradation, accompanied by disintegrated aggrecan and cell apoptosis and fibrosis, may lead to the pathological process and related clinical symptoms of IVDD [36]. In this study, the changes in ECM were detected by IHC staining, western blot, and RT-PCR. The results, both *in vivo* and *in vitro*, showed that PTH-treated mouse intervertebral discs or human NP cells promoted the secretion of collagen II and aggrecan, which had the most fundamental influence on the improvement of IVDD.

A study has shown that IL-6, IL-8, and prostaglandin E2 (PGE2) were increased in both normal and degenerative human intervertebral discs under lipopolysaccharide (LPS) stimulation [37]. In addition, substance P expression in cells extracted from degenerative discs upregulated IL-1*β*, IL-6, and IL-8 in both NP and AF, whereas RANTES and TNF-*α* were upregulated only in AF [38]. In this study, we injected PTH subcutaneously into the suspended mice and detected the changes in inflammatory factors in intervertebral discs by western blot, ELISA, and RT-PCR. The results showed that PTH could reduce the expression of IL-1*β*, IL-6, and TNF-*α* in the intervertebral discs, which confirmed the protective effect of PTH on intervertebral discs through its anti-inflammatory effect.

Oxidative stress is considered one of the main causes of molecular damage from exogenous and endogenous stress, under which the body produces a large amount of reactive oxygen species (ROS) [39]. ROS impairs disc structure and function by destroying lipids, DNA, and proteins. In addition, excess ROS production has been reported in degenerative intervertebral discs in rats. A study showed that hydrogen peroxide could downregulate the expression of collagen II and aggrecan in human and rat intervertebral disc cells [40]. In addition, proinflammatory cytokines that lead to ROS overproduction have been shown to inhibit matrix anabolism and increase the expression of proteases in human and rat intervertebral disc cells [41]. Our study found that the expressions of SOD1, SOD2, and CAT in the PTH-treated mouse intervertebral disc NP tissues were significantly increased compared with the model group, and their expressions also increased with increased PTH concentration, indicating that PTH can improve the oxidative stress state of intervertebral discs.

Cell loss due to necrosis and apoptosis occurs continuously throughout the life cycle and may play an important role in degenerative diseases [35]. Haschtmann et al. [42] found that necrocytosis and apoptosis caused by endplate rupture fracture may lead to degeneration in NP and AF by analysing the activity of lactate dehydrogenase and the expression of proapoptotic genes (such as caspase3) and proapoptotic proteins (such as FasL). Another study confirmed that under a pressure load of 1.0 MPa, not only did the NP cells undergo significant time-dependent apoptosis, but the pressure load also caused cell death [43]. Therefore, improving the activity of NP cells and inhibiting apoptosis are also key to reducing the degeneration of intervertebral discs. In both *in vivo* and *in vitro* studies, we have demonstrated that PTH-mediated proliferation of NP cells increased and apoptosis decreased. PCNA and Ki67 in the intervertebral discs of PTH-treated mice were significantly increased, and CCK8 assay and EdU cell proliferation assay verified the improvement on the proliferation of NP cells with PTH. These findings suggest that PTH significantly increases cellular activity, which has a fundamental effect on the maintenance of intervertebral disc stability.

The SHH signalling pathway plays a crucial part in the development of intervertebral discs. In the embryonic stage, a large number of SHH signalling molecules can be detected in notochord cells, and the SHH signalling pathway can lead to significant spinal dysplasia in mice [9, 10]. *In vivo*, the expression of SHH signalling pathway-related molecules (SHH, SMO, and GLI1) was significantly decreased in mice with TS, indicating that the degeneration of the mouse intervertebral disc tissue was related to the decreased activity of the SHH signalling pathway. However, the expression of SHH, SMO, and GLI1 increased in the discs treated with PTH, suggesting that the improvement caused by PTH is related to the activation of the SHH signalling pathway. *In vitro*, we verified the importance of the SHH signalling pathway for NP cells and found that PUA and CYA could, respectively, promote and inhibit the expression of collagen II in NP cells via using the SHH signalling agonist (PUA) and antagonist (CYA), indicating that CYA inhibited the SHH signalling pathway and weakened the protective effect of PTH on NP cells. Therefore, the interaction between the PTH signalling pathway and the SHH signalling pathway may be the key to alleviating IVDD.

In 1986, Montminy et al. [44] found that the expressions of many genes are regulated by cAMP, and the promoters of these genes contain a highly conservative 8-base palindrome sequence (5′-TGACGTCA-3′)—cAMP-response element (CRE). CREB, as the CRE binding protein, was identified. As a transcription factor, CREB is the final regulator of many important signalling pathways in normal cells, and the activation of its phosphorylation plays a role in biological processes such as inflammation, DNA repair, immune response, and cell cycle progression [45]. In the nervous system, CREB can regulate the expression of c-fos and brain-derived neurotrophic factor to achieve memory consolidation [46, 47].CREB has also been found to regulate the cell cycle by regulating cyclin A transcription at G1/S [48].

A substantial amount of evidence has shown that CREB is a PTH response transcription factor and plays a role in promoting the transcription of downstream target genes [49–51]. According to JASPAR database analysis, there was a possible binding site between CREB and the SHH promoter region. Through the ChIP and the dual Luc report assays, we have confirmed a new key interaction between CREB and SHH that allows CREB to bind to the promoter region of SHH and consequently promotes its transcription. In summary, PTH could activate SHH signalling by activating CREB for SHH transcription.

PTH and its analogues have been used in clinics and ongoing clinical trials to improve osteoporosis [52]. Zhou et al. [53] found that PTH (1–34) could delay osteoporosis by increasing new bone generation, thus effectively reducing adjacent segment disc degeneration of ovariectomized rats. This study has confirmed that PTH could also reduce inflammation and oxidative stress by promoting spinal stability and maintaining disc homeostasis via preserving the integrity of the ECM. Therefore, the dual protective effect of PTH on the vertebral body and intervertebral discs makes PTH a potential therapeutic agent for alleviating IVDD.

## 5. Conclusion

Although PTH is generally considered an important hormone that regulates calcium and phosphorus balance in the human body, we have focused on the effect of PTH on intervertebral discs and discovered that the PTH/PTH1R signalling pathway can mediate the SHH signalling pathway. In summary, exogenous PTH can effectively improve the microenvironment of the intervertebral disc by reducing inflammation, oxidative stress, and apoptosis and increasing the viability of NP cells. We have identified CREB as a mediator in intervertebral discs for PTH/PTH1R to attenuate the degeneration of NP cells.

## Figures and Tables

**Table 1:** Primers for RT-PCR.

Targets	Sense strand	Antisense strand
Collagen II	AAGTCCCGGTCCTCCTG	CGTCGTGCTGTCTCAAGG
Collagen X	GCCCTCAAGGTCCCATA	GCCGTTTTCACCTCTTCTT
IL-1*β*	AGTTGACGGACCCCAAA	TCTTGTTGATGTGCTGCTG
TNF-*α*	CGCTGAGGTCAATCTGC	GGCTGGGTAGAGAATGGA
IL-6	ACAGAAGGAGTGGCTAAGGA	AGGCATAACGCACTAGGTTT
SOD1	GGTTCCACGTCCATCAGT	ACATTGCCCAGGTCTCC
SOD2	ATTGACGTGTGGGAGCA	AATGTGGCCGTGAGTGA
CAT	CCTCGCAGAGACCTGATG	GCACCTGCTCCTTTTGC
PCNA	TTCACAAAAGCCACTCCAC	TGCCTAAGATGCTTCCTCA
Ki67	TTCCAAAGCTCACCAAGG	GCGGGATCGCATAGTTT
Caspase3	TGACTGGAAAGCCGAAAC	GCAAGCCATCTCCTCATC
Caspase9	AGCGATTCTGCCTTTCAC	TGGAGATTTTGTGGTCAGC
SMO	CTCCCCTTTGTCCTCACG	CCTCCCACAATAAGCACCA
GLI1	AGCAGGAATTGTTGTGGGA	TGAAGGGGCAGGATAGGA
GAPDH	TGTTTCCTCGTCCCGTAGA	ATCTCCACTTTGCCACTGC

## Data Availability

The raw data used to support the findings of this study are available from the corresponding author upon request.
